# Computational optimization for the deposition of bioconvection thin Oldroyd-B nanofluid with entropy generation

**DOI:** 10.1038/s41598-021-91041-5

**Published:** 2021-06-02

**Authors:** Auwalu Hamisu Usman, Noor Saeed Khan, Usa Wannasingha Humphries, Zafar Ullah, Qayyum Shah, Poom Kumam, Phatiphat Thounthong, Waris Khan, Attapol Kaewkhao, Amyia Bhaumik

**Affiliations:** 1 Department of Mathematics, Faculty of Science, King Mongkut’s University of Technology Thonburi (KMUTT), 126 Pracha-Uthit Road, Bang Mod, Thung Khru, Bangkok, 10140 Thailand; 2 KMUTTFixed Point Research Laboratory, Room SCL 802 Fixed Point Laboratory, Science Laboratory Building, Department of Mathematics, Faculty of Science, King Mongkut’s University of Technology Thonburi (KMUTT), 126 Pracha-Uthit Road, Bang Mod, Thung Khru, Bangkok, 10140 Thailand; 3 Department of Mathematical Sciences, Bayero University, Kano, Kano, 700241 Nigeria; 4 Department of Mathematics, Division of Science and Technology, University of Education, Lahore, 54770 Pakistan; 5 Center of Excellence in Theoretical and Computational Science (TaCS-CoE), Science Laboratory Building, Faculty of Science, King Mongkut’s University of Technology Thonburi (KMUTT), 126 Pracha-Uthit Road, Bang Mod, Thung Khru, Bangkok, 10140 Thailand; 6 Department of Basic Sciences and Islamiyat, University of Engineering and Technology, Peshawar, 25000 Khyber Pakhtunkhwa Pakistan; 7 Department of Mathematics and Statistics, Hazara University, Mansehra, 21120 Khyber Pakhtunkhwa Pakistan; 8 Faculty of Engineering, Lincoln University College (LUC), 1440 Lincoln, Malaysia; 9 Department of Medical Research, China Medical University Hospital, China Medical University, Taichung, 40402 Taiwan; 10 Department of Teacher Training in Electrical Engineering, Faculty of Technical Education, Renewable Energy Research Centre, King Mongkut’s University of Technology North Bangkok, 1518 Pracharat 1 Road, Bangsue, Bangkok, 10800 Thailand; 11 Research Center in Mathematics and Applied Mathematics, Department of Mathematics, Faculty of Science, Chiang Mai University, Chiang Mai, 50200 Thailand

**Keywords:** Engineering, Materials science, Mathematics and computing, Nanoscience and technology, Physics

## Abstract

The behavior of an Oldroyd-B nanoliquid film sprayed on a stretching cylinder is investigated. The system also contains gyrotactic microorganisms with heat and mass transfer flow. Similarity transformations are used to make the governing equations non-dimensional ordinary differential equations and subsequently are solved through an efficient and powerful analytic technique namely homotopy analysis method (HAM). The roles of all dimensionless profiles and spray rate have been investigated. Velocity decreases with the magnetic field strength and Oldroyd-B nanofluid parameter. Temperature is increased with increasing the Brownian motion parameter while it is decreased with the increasing values of Prandtl and Reynolds numbers. Nanoparticle’s concentration is enhanced with the higher values of Reynolds number and activation energy parameter. Gyrotactic microorganism density increases with bioconvection Rayleigh number while it decreases with Peclet number. The film size naturally increases with the spray rate in a nonlinear way. A close agreement is achieved by comparing the present results with the published results.

## Introduction

The progress in non-Newtonian liquids has a great deal of importance in projects and emerging developments. Magnetohydrodynamics (MHD) applied to electrically conductive fluids primarily concerned with the results that can be obtained from the connection between fluid motion with any external magnetic field current. Albano et al.^1^ reported that metallurgy (form control, homogenization, sample levitation material), molten steel flow, planetary science and astrophysics, fusion reactors are some of non-Newtonian main applications. Various of fluids commonly used in industrial applications like poultry, cement, polymers, chemical, fermentation cycles, geothermal pools, pore drying, heat insulation, improved oil regeneration, etc., are non-Newtonian in nature. Khan and Nadeem^2^ analyzed the non-Newtonian Maxwell nanofluid flow past a linear/exponential stretching sheet in rotating system with double stratification, Arrhenius activation energy, temperature dependent thermal conductivity and thermophoresis. They used the bvp4c Matlab to evaluate the coupled ordinary differential equations and showed that rotation and stretching have remarkable effect on the velocity and temperature profiles. Khan and Nadeem^3^ presented the heat and mass transfer time dependent two-dimensional flow of bio-convective Maxwell nanofluid over an exponentially stretching sheet with viscous dissipation, external magnetic field, multiple slip conditions and chemical reaction. Due to the special behaviors, the Oldroyd-B fluid model is very important among the rate type fluids. Khan et al.^4^ explored the two- dimensional radiative Oldroyd-B nanofluid in transient flow past a permeable convectively heated stretching surface with gyrotactic microorganisms to explore that for the higher values of retardation parameter, velocity increases and heat transfer decreases. Khan et al.^5^ investigated dynamics with Cattaneo–Christov heat and mass flux theory of bioconvection Oldroyd-B nanofluid. Khan et al.^6^ investigated for the rotating flow of an Oldroyd-B fluid for improved thermal conduction and developed mass diffusion models. More detail on non-Newtonian fluids can be seen in the references^7–26^.

The cooling of liquid is enhanced by the nano-sized particles whose diameter ranges from $1-100$ nm. These nanoparticles are added into to the base fluid which enhance the cooling process, due to its higher heat transfer coefficient as compared to the conventional liquids. This mixture is called nanofluid. Choi and Bestman^27^ introduced the concept of nanofluid at Agronne National Laboratory, USA. Nanotechnology is one of the most interesting field nowadays. It is interesting due to its vast applications in medicine, electronics, solar cells, food, fuel cells, batteries etc. In simple, nanotechnology has made its way to every branch. The enhancement of the thermal properties of the liquids can be made by either metals or by metal oxides. It is often a special type of fluid with higher thermal conductivity than conventional host fluids (such as motor oil, glycols, water, etc.). Nanoparticles include metals (for example, aluminum, copper, nickel) and other elements (for example, carbon nanotubes, graphene, silicon carbide, calcium carbonate, titanium, etc.) as well as oxides (for example, alumina, titanium, silicone, silicon carbide, silicone carbonate, silicone, etc.). Buongiorno^28^ implemented a second phase nanofluid model in the awake of these models. Ellahi et al.^29^ investigated the heated couple stress bi-phase fluid with spherical particles of metal Hafnium. In that paper the flow bounded by two parallel plates is caused by solely the influence of pressure gradient in an axial direction. More studies on nanofluids can be found in the references^30–45^.

Entropy optimization in terms of irreversibility rate was investigated using thermodynamic second law. Entropy augmentation is used to illustrate the quality of various contexts in advanced and composition applications. Entropy is derived from the Greek word entropia, which means "change" or "movement in the direction of." The calculation of entropy is important because it categorizes the parameters for energy loss. Bejan^46^ introduced the concept of an entropy optimization problem. Khan et al.^47^ investigated entropy optimization in MHD viscous fluid flow using a stretchable sheet. Khan and Ali^48^ provided the modeling and simulation of entropy generation in dissipative cross materials with quartic autocatalysis. Further studies about entropy generation may be read in the references^49–55^.

Thin film flow is an important subject of research. Thin film fluids are used to produce different heat exchangers and chemical tools and these applications require a complete understanding of the motion procedure. Thin film fluids applications also include wire and fiber coating, preparation of polymers, etc. This motion is attached to the manufacturing of different types of sheets, either metal or plastic. In recent years, some researchers have considered working on this type of flow. Ellahi et al.^56^ studied the thin film coating on multi-fluid flow of a rotating disk suspended with nano-size silver and gold particles. More studies in this regard can be found in the references^57–59^.

Among the most significant indicators where the species does not usually respond to the chemical reactions are related with Arrhenius activation energy. The term energy activation was initially proposed by Arrhenius^60^. However, the minimum energy required for the operation of chemical reactions molecules or atoms is defined as energy activation. Perhaps for the first time, Bestman^61^ identified a primary model consisting of a limiting layer of fluid flow problems due to binary chemical reactions with Arrhenius activation energy. The emphasis here is on the flow of a binary chemical reacting fluid with Arrhenius activating energy and convective boundary conditions. The purpose of this work is to discuss the effect of activation energy on fluid flow and binary chemical reactions. The effect of frictional heating on binary chemical reactions can significantly reduce undue surface reactions and, as a result, improve deposition. Further studies can be found in the references^62–64^.

In food industry and many physiological fluid flow problems, the density of motile gyrotactic microorganisms is significant and this density of motile microorganisms plays a vital role in fluid flow. Bioconvection phenomena is a common phenomenon usually occurs in suspensions due to the up swimming of microorganisms that are marginally with high density than water. If the upper surface of the suspensions gets so dense due to the proliferation of microorganisms, then it becomes porous and the microorganisms collapse to cause bioconvection. The concept explains the formation of impulsive patterns and dense streaming formed at the concurrent boundary of more autonomously propelled microorganisms, nanoparticles, and buoyant forces. However, some forms that may constitute parts of these microorganisms are gravitaxis (describe the swimming motion against gravity), gyrotaxis (describe the way the swimming is guided through a balance between the physical torques generated by viscous drag and by gravity operating on an asymmetric distribution of mass within the organism) or oxytaxis (describe the swimming along an oxygen gradient). Supporting gyrotactic microorganisms for nanofluid helps to convert the mass to mix micro-scales and to increase the stability of nanofluids particularly in micro-volumes. The analysis highlights the principle of nanofluid bioconvection. Several researchers have investigated its numerous effects on fluid flow including nanofluid gyrotactic microorganisms which plays a very important role in increasing the greenhouse effects. Ghorai and Hill^65^ have shown stability and growth within a deep cavity with free stress on the side walls in the presence of gyrotactic microorganisms. Chamkha et al.^66^ investigated the radiation effects of gyrotactic microorganisms on a vertical plate with fluid variability in temperature on natural bioconvection flows. Rashad et al.^67^ studied a mixed bioconvection nanofluid flow with gyrotactic microorganisms through a thin vertical cylindrical under closed saturated porous medium using the transient mixed boundary layer convection. Hady et al.^68^ presented the unsteady bioconvection thermal boundary layer nanofluid flow in the presence of gyrotactic microorganisms on a stretching plate and a vertical cone in porous medium. More studies on bioconvection can be found in the references^69–73^.

It is observed that due to stretching cylinder the flow receives adequate attention. Wang^74,75^ was the first to study the steady-state incompressible viscous fluid across the growing hollow cylinder. Bachok and Ishak^76^ examined and reported the numerical flow and thermal transfer solution for the stretching cylinder. Chuhan et al.^77^ investigated the effects of magnetohydrodynamics and thermal radiation on the movement of fluid past a porous stretching cylinder. Irfan et al.^78^ studied the motion of a nanofluid past a stretching cylinder with heat transfer and magnetic field.

Literature has several interesting studies on stretching cylinder like references^79,80^ which are followed by the present study. Spraying phenomena occurs in the analysis and design of coating processes. This paper is unique in the sense that it investigates the film deposition of a bioconvection Oldroy-B nanofluid containing motile gyrotactic microorganisms on a stretching cylinder. In the present article, the steady two-dimensional, incompressible radiative flow of the Oldroy-B axisymmetric sprayed thin film nanofluid past a stretching cylinder is analyzed. The fluid flow problem is governed by the partial differential equations and are converted into ordinary ones by means of suitable similarity variables. Initially, Liao presented HAM^81–83^ in 1992. The solution of this method is fast convergent. Due to its rapid convergence, various researchers^84–88^ have used HAM to solve their fluid flow problems. The computed results concerning the effects of all the related parameters on all the profiles are presented graphically.

## Problem formulation

**Figure 1:**
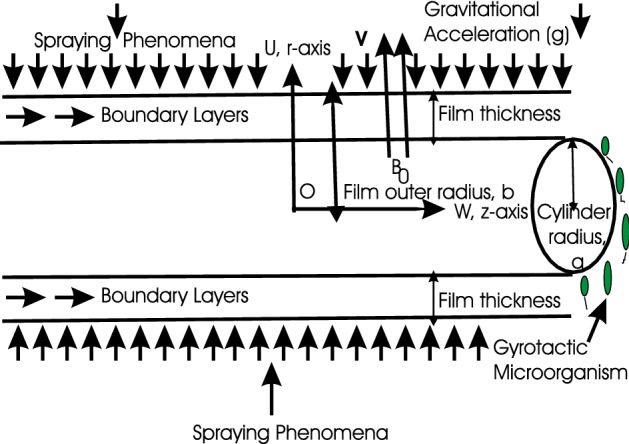
Geometry of the problem.

The steady, two-dimensional, and incompressible radiative Oldroyd-B and axisymmetric sprayed thin film nanofluid flow is considered past a stretching cylinder at $r = 0$. The flow is in the domain $r > 0$. The $z - axis$ is taken along the axis of cylinder and $r - axis$ is measured along the radial direction. The effects of the magnetic field are used in the direction of $r - axis$. Assuming induced magnetic field effects to be negligible. The expression $2cz$ is the surface velocity, where $z$ is the axial coordinate and $c$ is a proportional constant. As the material stretches, the cylinder's thickness decreases, but the cylinder's outer radius $a$ remains relatively constant. A radial axisymmetric spray with a $V$ velocity condenses as a film and is drawn in by the cylinder's outer surface (see Fig. 1).

The basic governing equations for the fluid flow are as ^56–59,74,75,79,80^:

1 $$\frac{\partial u}{{\partial r}} + \frac{u}{r} + \frac{\partial w}{{\partial z}} = 0,$$

2 $$\begin{gathered} u\frac{\partial w}{{\partial r}} + w\frac{\partial w}{{\partial z}} + k_{0} \left[ {2\frac{{\partial^{2} w}}{\partial r\partial z}wu + \frac{{\partial^{2} w}}{{\partial z^{2} }}w^{2} + \frac{{\partial^{2} w}}{{\partial r^{2} }}u^{2} } \right] = v_{f} \left[ {\frac{{\partial^{2} w}}{{\partial r^{2} }} + \frac{1}{r}\frac{\partial w}{{\partial r}}} \right] + \sigma B_{o}^{2} \left( { - w - k_{o} \frac{\partial w}{{\partial r}}u} \right) + \hfill \\ \frac{{\nu_{f} k_{1} }}{{\rho_{f} }}\left[ \begin{gathered} \frac{\partial w}{{\partial r}}\frac{u}{{r^{2} }} - \frac{\partial w}{{\partial z}}\frac{\partial w}{{\partial r}}\frac{1}{r} - 2\frac{\partial w}{{\partial r}}\frac{{\partial^{2} u}}{{\partial r^{2} }} + \frac{\partial w}{{\partial z}}\frac{{\partial^{2} w}}{{\partial r^{2} }}\frac{u}{r} + \frac{{\partial^{2} w}}{\partial r\partial z}\frac{w}{r} + \hfill \\ u\frac{{\partial^{2} w}}{{\partial r^{2} }} + w\frac{{\partial^{3} w}}{{\partial z\partial^{2} r}} - \frac{\partial u}{{\partial r}}\frac{\partial w}{{\partial r}}\frac{2}{r} - \frac{{\partial^{2} w}}{\partial r\partial z}\frac{\partial w}{{\partial r}} + \frac{{\partial^{{^{2} }} w}}{{\partial r^{2} }}\frac{u}{r} \hfill \\ \end{gathered} \right] + \left[ {\frac{1}{{\rho_{f} }}\left[ \begin{gathered} \left( {1 - C_{b} } \right)\rho_{f} \beta^{ * } \left( {T - T_{b} } \right) - \hfill \\ \left( {\rho_{p} - \rho_{f} } \right)\left( {C - C_{b} } \right) - \hfill \\ \left( {N - N_{b} } \right)\left( {\rho_{m} - \rho_{f} } \right) \hfill \\ \end{gathered} \right]g} \right], \hfill \\ \hfill \\ \end{gathered}$$

3 $$u\frac{\partial T}{{\partial r}} + w\frac{\partial T}{{\partial z}} = \alpha_{1} \left[ {\frac{{\partial^{2} T}}{{\partial r^{2} }} + \frac{1}{r}\frac{\partial T}{{\partial r}}} \right] + \frac{{\mu_{f} }}{{(\rho c_{p} )_{f} }}\left( {\frac{\partial w}{{\partial r}}} \right)^{2} + \tau \left[ {D_{B} \frac{\partial C}{{\partial r}}\frac{\partial T}{{\partial r}} + \frac{{D_{T} }}{{T_{b} }}\left( {\frac{\partial T}{{\partial r}}} \right)^{2} } \right] - \frac{1}{{(\rho c_{p} )_{f} }}\frac{{\partial (rq_{r} )}}{\partial r},$$

4 $$u\frac{\partial C}{{\partial r}} + w\frac{\partial C}{{\partial z}} = D_{B} \frac{1}{r}\frac{\partial }{\partial r}\left( {r\frac{\partial C}{{\partial r}}} \right) + \frac{{D_{T} }}{{T_{b} }}\frac{1}{r}\frac{\partial }{\partial r}\left( {r\frac{\partial T}{{\partial r}}} \right) - k_{r}^{2} \left( {C - C_{b} } \right)\left( {\frac{T}{{T_{b} }}} \right)^{m} \exp \left[ {\frac{{ - E_{a} }}{kT}} \right],$$

5 $$u\frac{\partial N}{{\partial r}} + w\frac{\partial N}{{\partial z}} + \frac{{bW_{c} }}{{(C_{w} - C_{b} )}}\left[ {\frac{\partial }{\partial r}\left( {N\frac{\partial C}{{\partial r}}} \right)} \right] = D_{m} \left( {\frac{{\partial^{2} N}}{{\partial r^{2} }}} \right) ,$$

6 $$\begin{gathered} w(z,r) = W_{w} (z) = 2cz,\,\,\,\,u(z,r) = U_{w} (z),\,\,\,T(z,r) = T_{w} (z) = T_{b} - T_{ref} \left[ {\frac{{cz^{2} }}{{v_{f} }}} \right], \hfill \\ {\text{C(}}z,r{)} = C_{w} (z) = C_{b} - C_{ref} \left[ {\frac{{cz^{2} }}{{v_{f} }}} \right]{, }\,N{(}z,r{)} = N_{w} (z) = N_{b} - N_{ref} \left[ {\frac{{cz^{2} }}{{v_{f} }}} \right]{\text{ at }}r = a, \hfill \\ \end{gathered}$$

7 $$\frac{\partial w}{{\partial r}} = 0,\frac{\partial \delta }{{\partial r}} = 0,\frac{\partial C}{{\partial r}} = 0,\frac{\partial T}{{\partial r}} = 0,\frac{\partial N}{{\partial r}} = 0, \, u = \frac{\partial \delta }{{\partial z}}{\text{ at }}r = b,$$

where $\delta$ is the film size.

According to the Rosseland approximation the thermally developed flow can be expressed as a modification^2^,

8 $$q_{r} = - \frac{{16\sigma^{**} T_{b}^{3} }}{{3k^{**} }}\frac{\partial T}{{\partial r}} \, {.}$$

Introducing the transformation for non-dimensionless functions $f,\theta ,\phi ,\chi$ and similarity variable $\zeta$ ^74,79^ as

9 $$\begin{gathered} \zeta = \left( \frac{r}{a} \right)^{2} , \, u = - ca\frac{f(\zeta )}{{\sqrt \zeta }}, \, w = 2czf^{\prime}(\zeta ), \, T(z) = T_{b} - T_{ref} \left[ {\frac{{cz^{2} }}{{v_{f} }}} \right]\theta {(}\zeta {)}, \hfill \\ N(z) = N_{b} - N_{ref} \left[ {\frac{{cz^{2} }}{{v_{f} }}} \right]\chi (\varsigma ),{\text{ C}}(z) = C_{b} - C_{ref} \left[ {\frac{{cz^{2} }}{{v_{f} }}} \right]\phi (\zeta ). \hfill \\ \end{gathered}$$

At the outer radius $b$ of the film thickness

10 $$\zeta = \left( \frac{b}{a} \right)^{2} = \beta_{1} .$$

Equation (1) is satisfied through Eqs. (9, 10) whereas Eqs. (2)–(7) have the following form

11 $$\begin{gathered} \frac{1}{{\text{Re}}}(2f^{\prime\prime} + 2\zeta f^{\prime\prime\prime}) - Mf^{\prime} + ff^{\prime\prime} - f^{{\prime}{2}} + \lambda_{1} \left( {4ff^{\prime}f^{\prime\prime} + \frac{1}{\zeta }f^{2} f^{\prime\prime} - 2f^{2} f^{\prime\prime\prime} - 2Mff^{\prime\prime}} \right) + \hfill \\ 2\lambda_{2} \left[ {2\zeta f^{\prime}f^{\prime\prime\prime} + \frac{2}{\zeta }ff^{\prime\prime} - ff^{\prime\prime\prime} + 2\zeta f^{{\prime\prime}{2}} - 2ff^{\prime}f^{\prime\prime\prime} - \frac{2}{\zeta }ff^{\prime}f^{\prime\prime}} \right] - Gr\theta + Gm\phi - Rb\chi = 0, \hfill \\ \end{gathered}$$

12 $$(2 + Rd)(\theta ^{\prime} + \zeta \theta ^{\prime\prime}) - Nb\phi ^{\prime}\theta ^{\prime} - Nt\theta ^{{\prime}{2}} + \frac{4\Pr Ec}{{\text{Re}}}\zeta f^{{\prime\prime}{2}} + Pr(f\theta ^{\prime} - 2f^{\prime}\theta ) = 0,$$

13 $$Sc(\phi ^{\prime} + \zeta \phi ^{\prime\prime}) + f\phi ^{\prime} - 2f^{\prime}\phi + Sc_{b} (\theta ^{\prime} + \zeta \theta ^{\prime\prime}) - \gamma_{1} \left( {\gamma_{2} - \theta_{w} \theta } \right)^{m} e^{{ - \left[ {\frac{E}{{\left( {\gamma_{2} - \theta_{w} \theta } \right)}}} \right]}} = 0 ,$$

14 $$2\zeta \chi ^{\prime\prime} + \chi ^{\prime} + Lb(f\chi ^{\prime} - 2f^{\prime}\chi ) + Pen_{1} (\phi ^{\prime} + 2\zeta \phi ^{\prime\prime}) - Pe\left[ {(\phi ^{\prime} + 2\zeta \phi ^{\prime\prime})\chi + 2\phi ^{\prime}\chi ^{\prime}} \right] = 0 ,$$

with boundary conditions

15 $$\begin{gathered} f = f^{{\prime}} = \theta = \phi = \chi = 1\,\,\,at\,\,\varsigma = 1 \hfill \\ f^{{\prime\prime}} = \theta^{{\prime}} = \phi^{{\prime}} = \chi^{{\prime}} = 0\,\,\,at\,\,\varsigma = \beta_{1}. \hfill \\ \end{gathered}$$

The dimensionless parameters are defined as

$$\begin{gathered} {\text{Re}} = \frac{{ca^{2} }}{{\nu_{f} }},M = \frac{{\sigma_{f} B_{0}^{2} }}{{2c\rho_{f} }},\lambda_{1} = \frac{{ck_{0} }}{{\rho_{f} }},\lambda_{2} = \frac{{k_{1} c}}{{\rho_{f} }},Gr = \frac{{g\beta^{*} (1 - C_{b} )(T_{w} - T_{b} )}}{{4c^{2} a}},Gm = \frac{{g(\rho_{p} - \rho_{f} )(C_{w} - C_{b} )}}{{4c^{2} \rho_{f} a}} \hfill \\ Rb = \frac{{g(\rho_{m} - \rho_{f} )(N_{w} - N_{b} )}}{{4c^{2} \rho_{f} a}},Rd = \frac{{32\sigma^{**} T_{\infty }^{3} }}{{3(\rho c_{p} )_{f} k^{**} \alpha_{1} }}, \, Nb = \frac{{\tau D_{B} (C_{w} - C_{b} )}}{{\alpha_{1} }}, \, Nt = \frac{{\tau D_{T} (T_{w} - T_{b} )}}{{\alpha_{1} }}, \, \Pr = \frac{{ca^{2} }}{{\alpha_{1} }} \hfill \\ \end{gathered}$$

16 $$Sc = \frac{{2D_{B} }}{{ca^{2} }}, \, Sc_{b} = \frac{{2D_{T} T_{ref} }}{{T_{b} ca^{2} C_{ref} }}, \, \gamma_{1} = \frac{{k_{r}^{2} }}{2c}, \, \gamma_{2} = \frac{{T_{w} }}{{T_{b} }},\theta_{w} = \frac{{T_{w} - T_{b} }}{{T_{b} }}, \, E_{1} = \frac{{E_{a} }}{{kT_{b} }}, \, Pe = \frac{{b_{1} W_{c} }}{{D_{m} }},Lb = \frac{{ca^{2} }}{{D_{m} }},{\text{ n}}_{1} = \frac{{N_{b} }}{{N_{ref} }}.$$

The shear stress on the surface of the outer film is zero i.e.

17 $$f^{\prime\prime}(\beta_{1} ) = 0.$$

And the shear stress on the cylinder is

18 $$\tau = \frac{{\rho_{f} v_{f} 4czf^{\prime\prime}(1)}}{a} = \frac{{4cz\mu_{f} f^{\prime\prime}(1)}}{a}.$$

The deposition velocity $V$ in terms of film thickness $\beta_{1}$ is given by

19 $$ca\frac{{f(\beta_{1} )}}{{\sqrt {\beta_{1} } }} = V.$$

Mass flux $m_{1}$ is another interesting quantity which in connection with the deposition per axial length is

20 $$m_{1} = 2\pi bV .$$

The normalized mass flux $m_{2}$ is

21 $$m_{2} = \frac{{m_{1} }}{{2\pi a^{2} c}} = \frac{{m_{1} }}{{4\pi v_{f} {\text{Re}} }} = f(\beta_{1} ) .$$

### Physical quantities

The physical quantities of interests are given as following.

### Skin friction coefficient

$$C_{f} = \frac{{2\tau_{rz} }}{{\rho_{f} (W_{w} )^{2} }}|_{r = a} ,\;{\text{where}}\;\tau_{rz} = \mu_{f} \left( {\frac{\partial w}{{\partial r}}} \right)_{r = a} ,$$

22 $$C_{f} = \frac{2}{{{\text{Re}}_{z}^{{\tfrac{1}{2}}} }}f^{\prime\prime}(1),\quad {\text{with}}\quad {\text{Re}}_{z} = \frac{{ca^{2} z}}{{v_{f} }},$$

### Nusselt number

$$Nu = \frac{{aq_{h} }}{{k(T_{w} - T_{b} )}}|_{r = a} ,\quad {\text{where}}\quad q_{h} = - k\frac{\partial T}{{\partial r}}|_{r = a} ,$$

23 $$Nu = - { 2}\theta ^{\prime}(1).$$

### Sherwood number

$$Sh = \frac{{aq_{m} }}{{D_{B} (C_{w} - C_{b} )}}|_{r = a} ,\quad {\text{where}}\quad q_{m} = - D_{B} \frac{\partial C}{{\partial r}}|_{r = a} ,$$

24 $$Sh = - { 2}\phi ^{\prime}(1).$$

### Local density motile flux

$$Sn = \frac{{q_{n} }}{{D_{n} (N_{w} - N_{b} )}}|_{r = a} ,\quad {\text{where}}\quad q_{n} = - D_{n} \frac{\partial N}{{\partial r}}|_{r = a} ,$$

25 $$Sn = - { 2}\chi ^{\prime}(1).$$

### Analysis of entropy generation

For the bio-nanofluid system, the irreversibility formulation is

26 $$\begin{gathered} E^{\prime\prime\prime}_{gen} = \frac{{\alpha_{1} }}{{T_{b}^{2} }}\left[ {1 + \frac{{16T_{1}^{3} \sigma^{*} }}{{K(T)k^{*} }}} \right]\left( {\frac{\partial T}{{\partial r}}} \right)^{2} + \frac{{\mu_{f} }}{{T_{b} }}\left( {\frac{\partial w}{{\partial r}}} \right)^{2} + \frac{RD}{{C_{b} }}\left( {\frac{\partial C}{{\partial r}}} \right)^{2} + \frac{RD}{{T_{b} }}\left( {\frac{\partial T}{{\partial r}}\frac{\partial C}{{\partial r}} + \frac{\partial C}{{\partial z}}\frac{\partial T}{{\partial z}}} \right)\,\, + \hfill \\ \frac{RD}{{N_{b} }}\left( {\frac{\partial N}{{\partial r}}} \right)^{2} + \frac{RD}{{T_{b} }}\left( {\frac{\partial T}{{\partial r}}\frac{\partial N}{{\partial r}} + \frac{\partial C}{{\partial z}}\frac{\partial N}{{\partial z}}} \right)\,\, + \frac{{\sigma_{f} B_{o}^{2} w^{2} }}{{T_{b} }}, \hfill \\ \end{gathered}$$

where *R* denotes the ideal gas constant and *D* represents the diffusivity.

In Eq. (26), the first term represents the irreversibility due to heat transfer, the second term is entropy generation due to viscous dissipation and third to six terms are irreversibility due to diffusion effect. The seventh term stands for the entropy generation due to magnetic field. The characteristic entropy generation rate is

27 $$E^{\prime\prime\prime}_{0} = \frac{{\alpha_{1} \left( {T_{a} - T_{b} } \right)^{2} }}{{T_{b}^{2} }}.$$

Notice that irreversibility $N_{G} \left( \varsigma \right)$ in scaled form is

28 $$N_{G} (\varsigma ) = \frac{{E^{\prime\prime\prime}_{gen} }}{{E^{\prime\prime\prime}_{0} }} .$$

Using Eqs. (9, 10), dimensional Eq. (28) converted into the following dimensionless form

29 $$\begin{gathered} N_{G} (\varsigma ) = \frac{4}{{a^{2} }}\left( {1 + \frac{4}{3}Rd} \right)\,\theta^{{\prime}{2}} + \frac{Br}{{\theta_{w}^{2} }}\,f^{{\prime}{2}} + B_{1} \left( {\frac{{\phi_{w} }}{{\theta_{w} }}} \right)^{2} \phi^{2} + a^{2} B_{1} \frac{{\phi_{w} }}{{\theta_{w} }}\phi^{\prime}\theta^{\prime}\, + B_{1} \frac{{\phi_{w} }}{{\theta_{w} }}\phi \theta + \,B_{2} \left( {\frac{{\chi_{w} }}{{\theta_{w} }}} \right)^{2} \chi^{{\prime}{2}} \, \hfill \\ + \,\,a^{2} B_{2} \frac{{\chi_{w} }}{{\theta_{w} }}\,\chi^{\prime}\theta^{\prime} + B_{3} \frac{{\phi_{w} \chi_{w} }}{{\theta_{w}^{2} }}\phi \theta + Mf^{{\prime}{2}} , \hfill \\ \end{gathered}$$

where $N_{G}$ represents the entropy generation rate, $Br = \frac{{4c^{2} \mu }}{{\alpha_{1} (T_{w} - T_{b} )}},$ $B_{1} = \frac{{4RDC_{b} }}{{\alpha_{1} }},$ $B_{2} = \frac{{4RDN_{b} }}{{\alpha_{1} }},$ $B_{3} = \frac{{4RDC_{b} N_{b} }}{{\alpha_{1} T_{b} }},$ $M = \frac{{4c^{2} a^{2} \sigma_{nf} B_{0}^{2} }}{{\alpha_{1} }}$ are respectively the Brinkman number, diffusivity constant parameters due to nanoparticle and gyrotactic microorganism concentration and magnetic field parameter. $\,\,\theta_{w} = \frac{{\left( {T_{a} - T_{b} } \right)}}{{T_{b} }}$,$\,\phi_{w} = \frac{{\left( {C_{a} - C_{b} } \right)}}{{C_{b} }}$,$\,\,\chi_{w} = \frac{{N_{a} - N_{b} }}{{N_{b} }}$ are respectively the dimensionless heat, nanoparticle concentration and microorganism concentration ratio variables.

## Solution of the problem by homotopy analysis method (HAM)

Taking the initial guesses and the linear operators as

30 $$f_{o} (\zeta ) = (1 - e^{ - \zeta } ),\theta_{o} = e^{ - \zeta } ,\phi_{o} = e^{ - \zeta } ,\chi_{o} = e^{ - \zeta } ,$$

31 $$L_{f} = f^{\prime\prime\prime} - f^{\prime}, \, L_{\theta } = \theta ^{\prime\prime} - \theta , \, L_{\phi } = \phi ^{\prime\prime} - \phi ,{\text{ and }}L_{\chi } = \chi ^{\prime\prime} - \chi ,$$

satisfying the properties as given below

32 $$L_{f} \left[ {C_{1} + C_{2} e^{\zeta } + C_{3} e^{ - \zeta } } \right] = 0,$$

33 $$L_{\theta } \left[ {C_{4} e^{\zeta } + C_{5} e^{ - \zeta } } \right] = 0,$$

34 $$L_{\phi } \left[ {C_{6} e^{\zeta } + C_{7} e^{ - \zeta } } \right] = 0,$$

35 $$L_{\chi } \left[ {C_{8} e^{\zeta } + C_{9} e^{ - \zeta } } \right] = 0,$$

where $\left\{ {C_{i} } \right\}_{i = 1}^{9}$ are the arbitrary constants.

The zeroth order form of the problems are given as

36 $$(1 - p)L_{f} \left[ {f(\zeta ,p) - f_{o} (\zeta )} \right] = p\hbar_{f} N_{f} \left[ {f(\zeta ,p),\theta (\zeta ,p),\phi (\zeta ,p),\chi (\zeta ,p)} \right] ,$$

37 $$(1 - p)L_{\theta } \left[ {\theta (\zeta ,p) - \theta_{o} (\zeta )} \right] = p\hbar_{\theta } N_{\theta } \left[ {f(\zeta ,p),\theta (\zeta ,p),\phi (\zeta ,p)} \right] ,$$

38 $$(1 - p)L_{\phi } \left[ {\phi (\zeta ,p) - \phi_{o} (\zeta )} \right] = p\hbar_{\phi } N_{\phi } \left[ {f(\zeta ,p),\theta (\zeta ,p),\phi (\zeta ,p)} \right],$$

39 $$(1 - p)L_{\chi } \left[ {\chi (\zeta ,p) - \chi_{o} (\zeta )} \right] = p\hbar_{\chi } N_{\chi } \left[ {f(\zeta ,p),\theta (\zeta ,p),\phi (\zeta ,p),\chi (\zeta ,p)} \right] ,$$

40 $$\begin{gathered} f(1,p) = 1,f^{\prime}(\beta_{1} ,p) = 0,f^{\prime}(1,p) = 1,\theta (1,p) = 1,\theta (\beta_{1} ,p) = 0, \hfill \\ \phi (1,p) = 1,\phi (\beta_{1} ,p) = 0,\chi (1,p) = 1,\chi (\beta_{1} ,p) = 0, \hfill \\ \end{gathered}$$

where $p$ is an embedding parameter in this case and $\hbar_{f} ,\hbar_{\theta } ,\hbar_{\phi } ,\hbar_{\chi }$ are the non-zero auxiliary parameters. $N_{f} ,N_{\theta } ,N_{\phi } ,N_{\chi }$ represent the none-linear operators and can be obtained through Eqs. (11)–(14) as follows

41 $$\begin{gathered} N_{f} \left[ {f(\zeta ,p),\theta (\zeta ,p),\phi (\zeta ,p),\chi (\zeta ,p)} \right] = \frac{2}{{\text{Re}}}\left( {\frac{{\partial^{2} f(\zeta ,p)}}{{\partial \zeta^{2} }} + \zeta \frac{{\partial^{3} f(\zeta ,p)}}{{\partial \zeta^{3} }}} \right) - M\frac{\partial f(\zeta ,p)}{{\partial \zeta }} \hfill \\ + f(\zeta ,p)\frac{{\partial^{2} f(\zeta ,p)}}{{\partial \zeta^{2} }} - \left( {\frac{\partial f(\zeta ,p)}{{\partial \zeta }}} \right)^{2} + \lambda_{1} \left( \begin{gathered} 4f(\zeta ,p)\frac{\partial f(\zeta ,p)}{{\partial \zeta }}\frac{{\partial^{3} f(\zeta ,p)}}{{\partial \zeta^{3} }} + \frac{1}{\zeta }f^{2} (\zeta ,p)\frac{{\partial^{2} f(\zeta ,p)}}{{\partial \zeta^{2} }} \hfill \\ - f^{2} (\zeta ,p)\frac{{\partial^{3} f(\zeta ,p)}}{{\partial \zeta^{3} }} - Mf(\zeta ,p)\frac{{\partial^{2} f(\zeta ,p)}}{{\partial \zeta^{2} }} \hfill \\ \end{gathered} \right) \hfill \\ + 2\lambda_{2} \left( \begin{gathered} 2\zeta \frac{\partial f(\zeta ,p)}{{\partial \zeta }}\frac{{\partial^{3} f(\zeta ,p)}}{{\partial \zeta^{3} }} + \frac{2}{\zeta }f(\zeta ,p)\frac{{\partial^{2} f(\zeta ,p)}}{{\partial \zeta^{2} }} - f(\zeta ,p)\frac{{\partial^{3} f(\zeta ,p)}}{{\partial \zeta^{3} }} + 2\zeta \left( {\frac{{\partial^{2} f(\zeta ,p)}}{{\partial \zeta^{2} }}} \right)^{2} \hfill \\ - 2f(\zeta ,p)\frac{\partial f(\zeta ,p)}{{\partial \zeta }}\frac{{\partial^{3} f(\zeta ,p)}}{{\partial \zeta^{3} }} - \frac{2}{\zeta }f(\zeta ,p)\frac{\partial f(\zeta ,p)}{{\partial \zeta }}\frac{{\partial^{3} f(\zeta ,p)}}{{\partial \zeta^{3} }} \hfill \\ \end{gathered} \right) \hfill \\ - Gr\theta (\zeta ,p) + Gm\phi (\zeta ,p) - Rb\chi (\zeta ,p), \hfill \\ \end{gathered}$$

42 $$\begin{gathered} N_{\theta } \left[ {f(\zeta ,p),\theta (\zeta ,p),\phi (\zeta ,p)} \right] = (2 + Rd)\left( {\frac{\partial \theta (\zeta ,p)}{{\partial \zeta }} + \zeta \frac{{\partial^{2} \theta (\zeta ,p)}}{{\partial \zeta^{2} }}} \right) \hfill \\ - Nb\frac{\partial \phi (\zeta ,p)}{{\partial \zeta }}\frac{\partial \theta (\zeta ,p)}{{\partial \zeta }} - Nt\left( {\frac{\partial \theta (\zeta ,p)}{{\partial \zeta }}} \right)^{2} + \Pr \left( {f(\zeta ,p)\frac{\partial \theta (\zeta ,p)}{{\partial \zeta }} - 2\theta (\zeta ,p)\frac{\partial f(\zeta ,p)}{{\partial \zeta }}} \right) \hfill \\ \end{gathered}$$

$$Sc(\phi ^{\prime} + \zeta \phi ^{\prime\prime}) + f\phi ^{\prime} - 2f^{\prime}\phi + Sc_{b} (\theta ^{\prime} + \zeta \theta ^{\prime\prime}) - \gamma_{1} \left( {\gamma_{2} - \theta_{w} \theta } \right)^{m} e^{{ - \left[ {\frac{E}{{\left( {\gamma_{2} - \theta_{w} \theta } \right)}}} \right]}} = 0,$$

43 $$\begin{gathered} N_{\phi } \left[ {f(\zeta ,p),\theta (\zeta ,p),\phi (\zeta ,p)} \right] = Sc\left( {\frac{\partial \phi (\zeta ,p)}{{\partial \zeta }} + \zeta \frac{{\partial^{2} \phi (\zeta ,p)}}{{\partial \zeta^{2} }}} \right) + f(\zeta ,p)\frac{\partial \phi (\zeta ,p)}{{\partial \zeta }} \hfill \\ - 2\phi (\zeta ,p)\frac{\partial f(\zeta ,p)}{{\partial \zeta }} + Sc_{b} \left( {\frac{\partial \theta (\zeta ,p)}{{\partial \zeta }} + \zeta \frac{{\partial^{2} \theta (\zeta ,p)}}{{\partial \zeta^{2} }}} \right) - \gamma_{1} \left( {\gamma_{2} - \theta_{w} \theta (\zeta ,p)} \right)^{m} epx\left[ {\frac{ - E}{{\left( {\gamma_{2} - \theta_{w} \theta (\zeta ,p)} \right)}}} \right] \hfill \\ \end{gathered}$$

44 $$\begin{gathered} N_{\chi } \left[ {f(\zeta ,p),\phi (\zeta ,p),\chi (\zeta ,p)} \right] = 2\zeta \frac{{\partial^{2} \chi (\zeta ,p)}}{{\partial \zeta^{2} }} + \frac{\partial \chi (\zeta ,p)}{{\partial \zeta }} \hfill \\ + Lb\left( {f(\zeta ,p)\frac{\partial \chi (\zeta ,p)}{{\partial \zeta }} - 2\chi (\zeta ,p)\frac{\partial f(\zeta ,p)}{{\partial \zeta }}} \right) + Pen_{1} \left( {\frac{\partial \phi (\zeta ,p)}{{\partial \zeta }} + 2\zeta \frac{{\partial^{2} \phi (\zeta ,p)}}{{\partial \zeta^{2} }}} \right). \hfill \\ - Pe\left[ {\left( {\frac{\partial \phi (\zeta ,p)}{{\partial \zeta }} + 2\zeta \frac{{\partial^{2} \phi (\zeta ,p)}}{{\partial \zeta^{2} }}} \right)\chi (\zeta ,p) + 2\frac{\partial \phi (\zeta ,p)}{{\partial \zeta }}\frac{\partial \chi (\zeta ,p)}{{\partial \zeta }}} \right] \hfill \\ \end{gathered}$$

For $p = 0$ and $p = 1$, the following results are obtained

45 $$\begin{gathered} f(\zeta ,0) = f_{0} (\zeta ),\theta (\zeta ,0) = \theta_{0} (\zeta ),\phi (\zeta ,0) = \phi_{0} (\zeta ),\chi (\zeta ,0) = \chi_{0} (\zeta ), \hfill \\ f(\zeta ,1) = f(\zeta ),\theta (\zeta ,1) = \theta (\zeta ),\phi (\zeta ,1) = \phi (\zeta ),\chi (\zeta ,1) = \chi (\zeta ). \hfill \\ \end{gathered}$$

Obviously, when $p$ is increased from $0{\text{ to }}1$, then $f(\zeta ,p),\theta (\zeta ,p),\phi (\zeta ,p),\chi (\zeta ,p)$ vary from $f_{o} (\zeta ),\theta_{o} (\zeta ),\phi_{o} (\zeta ),\chi_{o} (\zeta )$ to $f(\zeta ),\theta (\zeta ),\phi (\zeta ),\chi (\zeta )$. Through Taylor’s series expansion, the expressions in Eq. (45) become as the following

46 $$f(\zeta ,p) = f_{o} (\zeta ) + \sum\limits_{m = 1}^{\infty } {f_{m} (\zeta )p^{m} ,f_{m} (\zeta ) = \frac{1}{m!}\frac{{\partial^{m} f(\zeta ,p)}}{{\partial \zeta^{m} }}} |_{p = 0} ,$$

47 $$\theta (\zeta ,p) = \theta_{o} (\zeta ) + \sum\limits_{m = 1}^{\infty } {\theta_{m} (\zeta )p^{m} ,\theta_{m} (\zeta ) = \frac{1}{m!}\frac{{\partial^{m} \theta (\zeta ,p)}}{{\partial \zeta^{m} }}} |_{p = 0} ,$$

48 $$\phi (\zeta ,p) = \phi_{o} (\zeta ) + \sum\limits_{m = 1}^{\infty } {\phi_{m} (\zeta )p^{m} ,\phi_{m} (\zeta ) = \frac{1}{m!}\frac{{\partial^{m} \phi (\zeta ,p)}}{{\partial \zeta^{m} }}} |_{p = 0} ,$$

49 $$\chi (\zeta ,p) = \chi_{o} (\zeta ) + \sum\limits_{m = 1}^{\infty } {\chi_{m} (\zeta )p^{m} ,\chi_{m} (\zeta ) = \frac{1}{m!}\frac{{\partial^{m} \chi (\zeta ,p)}}{{\partial \zeta^{m} }}} |_{p = 0} .$$

The convergence of the series in Eqs. (46)–(49) depend strongly upon $\hbar_{f} ,\hbar_{\theta } ,\hbar_{\phi } ,\hbar_{\chi }$. By considering that $\hbar_{f} ,\hbar_{\theta } ,\hbar_{\phi } ,\hbar_{\chi }$ are selected properly so that the series in Eqs. (46)–(49) converge at $p = 1$, then the following simplifications are achieved

50 $$f(\zeta ) = f_{o} (\zeta ) + \sum\limits_{m = 1}^{\infty } {f_{m} (\zeta )}$$

51 $$\theta (\zeta ) = \theta_{o} (\zeta ) + \sum\limits_{m = 1}^{\infty } {\theta_{m} (\zeta )}$$

52 $$\phi (\zeta ) = \phi_{o} (\zeta ) + \sum\limits_{m = 1}^{\infty } {\phi_{m} (\zeta )}$$

53 $$\chi (\zeta ) = \chi_{o} (\zeta ) + \sum\limits_{m = 1}^{\infty } {\chi_{m} (\zeta )}$$

The result of the problems at order $m$ deformation can be constructed as follow

54 $$L_{f} \left[ {f_{m} (\zeta ) - \eta_{m} f_{m - 1} (\zeta )} \right] = \hbar_{f} R_{f}^{m} (\zeta )$$

55 $$L_{\theta } \left[ {\theta_{m} (\zeta ) - \eta_{m} \theta_{m - 1} (\zeta )} \right] = \hbar_{\theta } R_{\theta }^{m} (\zeta )$$

56 $$L_{\phi } \left[ {\phi_{m} (\zeta ) - \eta_{m} \phi_{m - 1} (\zeta )} \right] = \hbar_{\phi } R_{\phi }^{m} (\zeta )$$

57 $$L_{\chi } \left[ {\chi_{m} (\zeta ) - \eta_{m} \chi_{m - 1} (\zeta )} \right] = \hbar_{\chi } R_{\chi }^{m} (\zeta )$$

58 $$\begin{gathered} f_{m} (1) = f^{\prime}(1) = f^{\prime}(\beta_{1} ) = 0,\theta_{m} (1) = \theta_{m} (\beta_{1} ) = 0, \hfill \\ \phi_{m} (1) = \phi_{m} (\beta_{1} ) = 0,\chi_{m} (1) = \chi_{m} (\beta_{1} ) = 0 \hfill \\ \end{gathered}$$

where $R_{f}^{m} (\zeta ),R_{\theta }^{m} (\zeta ),R_{\phi }^{m} (\zeta ) \,$ and $R_{\chi }^{m} (\zeta )$ can be calculated as

59 $$\begin{gathered} R_{f}^{m} (\zeta ) = \tfrac{2}{{\text{Re}}}\left( {f_{{_{m - 1} }}^{{\prime\prime}} (\zeta ) + \zeta f_{m - 1}^{{\prime\prime\prime}} (\zeta )} \right) - Mf_{m - 1}^{{\prime}} + \sum\limits_{k = 0}^{m - 1} {f_{m - 1 - k} f_{{_{k} }}^{{\prime\prime}} (\zeta )} - \sum\limits_{k = 0}^{m - 1} {f_{m - 1 - k}^{{\prime}} f_{{_{k} }}^{{\prime}} (\zeta )} \hfill \\ + \lambda_{1} \left( \begin{gathered} 4\sum\limits_{k = 0}^{m - 1} {\left( {\sum\limits_{r = 0}^{k} {f_{m - 1 - k} f_{{_{k - r} }}^{{\prime}} (\zeta )} } \right)} f_{{_{r} }}^{{\prime\prime}} (\zeta ) + \frac{1}{\zeta }\sum\limits_{k = 0}^{m - 1} {\left( {\sum\limits_{r = 0}^{k} {f_{m - 1 - k} f_{k - r} (\zeta )} } \right)} f_{{_{r} }}^{{\prime\prime\prime}} (\zeta ) \hfill \\ - 2\sum\limits_{k = 0}^{m - 1} {\left( {\sum\limits_{r = 0}^{k} {f_{m - 1 - k} f_{k - r} (\zeta )} } \right)} f_{{_{r} }}^{{\prime\prime\prime}} (\zeta ) - 2M\sum\limits_{k = 0}^{m - 1} {f_{m - 1 - k} f_{{_{k} }}^{{\prime\prime}} (\zeta )} \hfill \\ \end{gathered} \right) \hfill \\ + 2\lambda_{2} \left[ \begin{gathered} 2\zeta \sum\limits_{k = 0}^{m - 1} {f_{{_{m - 1 - k} }}^{{\prime}} (\zeta )f_{{_{k} }}^{{\prime\prime}^{\prime}} (\zeta )} \frac{2}{\zeta }\sum\limits_{k = 0}^{m - 1} {f_{m - 1 - k} f_{{_{k} }}^{{\prime\prime}} (\zeta )} - \sum\limits_{k = 0}^{m - 1} {f_{m - 1 - k} f_{{_{k} }}^{{\prime}} (\zeta )} \hfill \\ + 2\sum\limits_{k = 0}^{m - 1} {f_{{_{m - 1 - k} }} (\zeta )f_{{_{k} }}^{{\prime\prime}} (\zeta ) - 2\sum\limits_{k = 0}^{m - 1} {\left( {\sum\limits_{r = 0}^{k} {f_{m - 1 - k} f^{\prime}_{k - r} (\zeta )} } \right)} f_{{_{r} }}^{{\prime\prime\prime}} (\zeta ) - \frac{2}{\zeta }\sum\limits_{k = 0}^{m - 1} {\left( {\sum\limits_{r = 0}^{k} {f_{m - 1 - k} f^{\prime}_{k - r} (\zeta )} } \right)} f_{{_{r} }}^{{\prime\prime}} (\zeta )} \hfill \\ \end{gathered} \right] \hfill \\ - Gr\theta_{m} (\zeta ) + Gm\phi_{m} (\zeta ) - Rb\chi_{m} (\zeta ) \hfill \\ \end{gathered}$$

60 $$\begin{gathered} R_{\theta }^{m} (\zeta ) = (2 + Rd)\left( {\theta_{m - 1}^{{\prime}} (\zeta ) + \zeta \theta_{m - 1}^{{\prime\prime}} (\zeta )} \right) - Nb\sum\limits_{k = 0}^{m - 1} {\phi_{{_{m - 1 - k} }}^{{\prime}} \theta_{k}^{{\prime}} } \hfill \\ - Nt\sum\limits_{k = 0}^{m - 1} {\theta_{{_{m - 1 - k} }}^{{\prime}} \theta_{k}^{{\prime}} } + \Pr \left( {\sum\limits_{k = 0}^{m - 1} {f_{m - 1 - k} \theta_{k}^{{\prime}} } - 2\sum\limits_{k = 0}^{m - 1} {\phi_{{_{m - 1 - k} }}^{{\prime}} \theta_{k} } } \right) \hfill \\ \end{gathered}$$

61 $$\begin{gathered} R_{\phi }^{m} (\zeta ) = Sc\left( {\phi_{m - 1}^{{\prime}} (\zeta ) + \zeta \phi_{m - 1}^{{\prime\prime}} (\zeta )} \right) + \sum\limits_{k = 0}^{m - 1} {\phi_{{_{m - 1 - k} }}^{{\prime}} (\zeta )f_{k} (\zeta )} - 2\sum\limits_{k = 0}^{m - 1} {f_{{_{m - 1 - k} }}^{{\prime}} (\zeta )\phi_{k} (\zeta )} \hfill \\ + Sc_{b} \left( {\theta_{m - 1}^{{\prime}} (\zeta ) + \zeta \theta_{m - 1}^{{\prime\prime}} (\zeta )} \right) - \gamma_{1} \left( {\gamma_{2} - \theta_{w} \theta_{m} (\zeta )} \right)^{m} epx\left[ {\frac{ - E}{{\left( {\gamma_{2} - \theta_{w} \theta_{m} (\zeta )} \right)}}} \right] \hfill \\ \end{gathered}$$

62 $$\begin{gathered} R_{\chi }^{m} (\zeta ) = 2\zeta \chi_{m - 1}^{{\prime\prime}} (\zeta ) + \chi_{m - 1}^{{\prime}} (\zeta ) + Lb\sum\limits_{k = 0}^{m - 1} {\chi_{{_{m - 1 - k} }}^{{\prime}} (\zeta )f_{k} (\zeta )} \hfill \\ - 2Lb\sum\limits_{k = 0}^{m - 1} {f_{{_{m - 1 - k} }}^{{\prime}} (\zeta )\chi_{k} (\zeta )} + Pen_{1} \left( {\phi_{m - 1}^{{\prime}} (\zeta ) + \zeta \phi_{m - 1}^{{\prime\prime}} (\zeta )} \right) \hfill \\ - Pe\left[ {\sum\limits_{k = 0}^{m - 1} {\phi_{m - 1 - k}^{{\prime}} (\zeta )\chi_{k} (\zeta )} + \zeta \sum\limits_{k = o}^{m - 1} {\phi_{m - 1 - k}^{{\prime\prime}} (\zeta )\chi_{k} } + 2\sum\limits_{k = 0}^{m - 1} {\phi_{m - 1 - k}^{{\prime}} \chi_{k}^{{\prime}} } (\zeta )} \right] \hfill \\ \end{gathered}$$

$$\eta_{m} = \left\{ \begin{gathered} 0,\,\,\,\,m \le 1 \hfill \\ 1,\,\,\,\,m > 1 \hfill \\ \end{gathered} \right\}$$.

The general solutions are

63 $$f_{m} (\zeta ) = f_{m}^{*} (\zeta ) + C_{1} + C_{2} e^{\zeta } + C_{3} e^{ - \zeta }$$

64 $$\theta_{m} (\zeta ) = \theta_{m}^{*} (\zeta ) + C_{4} e^{\zeta } + C_{5} e^{ - \zeta }$$

65 $$\phi_{m} (\zeta ) = \phi_{m}^{*} (\zeta ) + C_{6} e^{\zeta } + C_{7} e^{ - \zeta }$$

66 $$\chi_{m} (\zeta ) = \chi_{m}^{*} (\zeta ) + C_{8} e^{\zeta } + C_{9} e^{ - \zeta }$$

in which $f_{m}^{*} (\zeta ),\theta_{m}^{*} (\zeta ),\phi_{m}^{*} (\zeta ),\chi_{m}^{*} (\zeta )$ are the special solutions.

## Results and discussion

**Figure 2:**
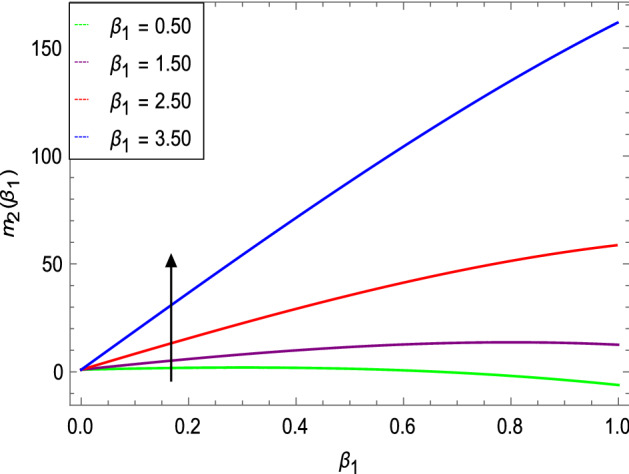
Spray rate as a function of *β*_1_.

The dynamics of an Oldroyd-B nanoliquid coolant and shielding paint or film sprayed on a stretching cylinder is studied. The normalized spray rate $m_{2}$ which is functionally correlated with the film size is shown in Fig. 2. The film size naturally increases with the spray rate at once, but not in a linear fashion. If the spray is not uniform, the film's outer surface may be affected. It's interesting to note that the spray rate increases the thickness of the film in a non-linear way. The spray deposits an Oldroyd-B nanoliquid film on the stretching cylinder, which can be used to cool the extruded material to promote solidification via a water bath or coolant spraying. Spraying also improves cooling because it creates a thinner boundary layer.

**Figure 3:**
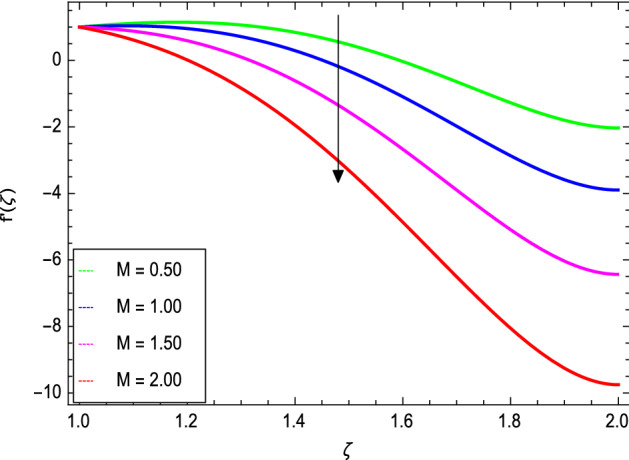
*f*′(ζ) as a function of *M*.

**Figure 4:**
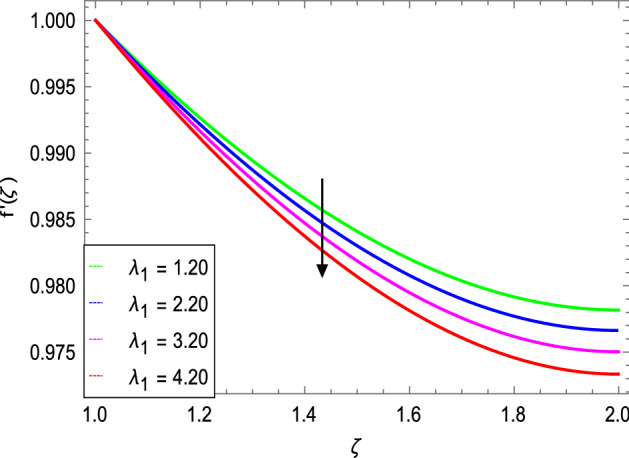
*f*′(ζ) as a function of *λ*_*1*_.

**Figure 5:**
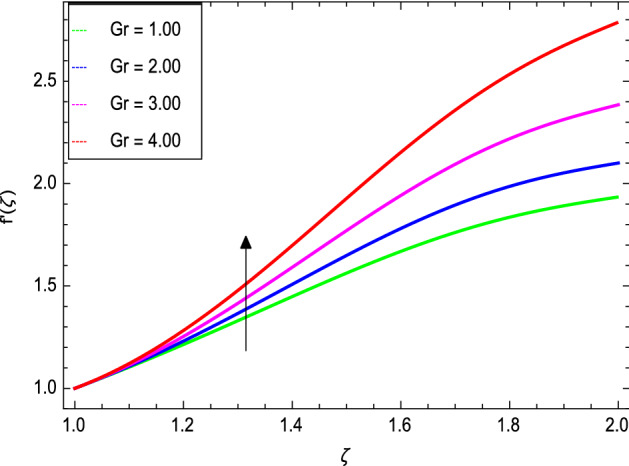
*f*′(ζ) as a function of *Gr*.

**Figure 6:**
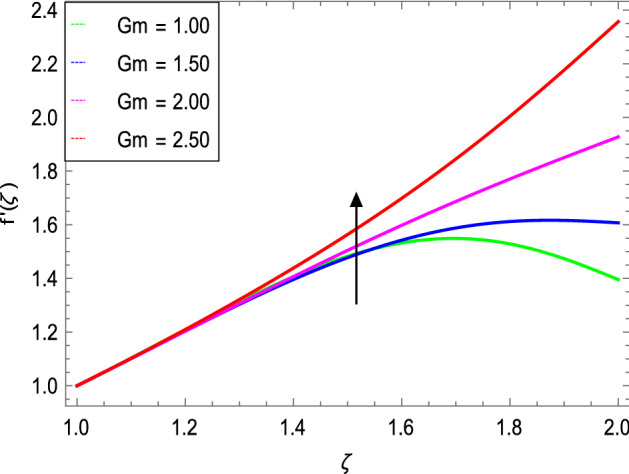
*f*′(ζ) as a function of *Gm*.

**Figure 7:**
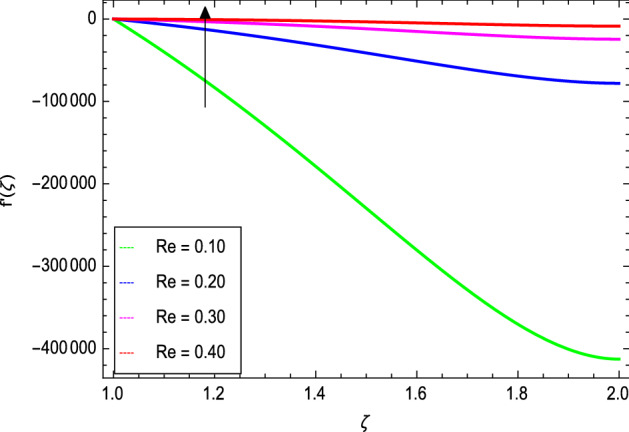
*f*′(ζ) as a function of *Re*.

Figures 3 and 4 depict the effect of the magnetic field ${\text{M}}$ and Oldroyd-B nanofluid parameter $\lambda_{1}$ on velocity profile. Figure 3 shows that the velocity decreases as the magnetic field parameter increases. In general, when a magnetic field is applied to a conduction-capable fluid flow, the momentum boundary layer becomes thin. The reason for this is that during this process, resistance forces known as Lorentz forces are produced, which have a negative impact on fluid flow. This force tends to slow the velocity of the nanofluid as it passes through the vertical surface. Figure 4 demonstrates that increasing the value of $\lambda_{1}$ decreases the velocity and hence momentum boundary layer thickness decreases. Thermal Grashof number $Gr$ and solutal Grashof number $Gm$ effects on the velocity profile are shown in Figs. 5 and 6. The graphs show that the velocity is increased with $Gr$ and $Gm$ due to the dominant effects of the buoyancy force in the central region and generates changes in the velocity and high viscous effects across the walls. As a result, when $Gm$ increases, the concentration of the liquid film increases directly and hence the viscosity increases. Figure 7 shows the effects of Reynolds number ${\text{Re}}$ on the velocity profile. The velocity is enhanced with the Reynolds number. The reason is that as the Reynolds number increases, the inertial force overcomes the flow regarding the viscous forces. High viscous forces are highly resistive to the fluid flow and with strong inertial forces, the flow of the boundary layer decreases. When ${\text{Re}}$ is small, then it means there exists small inertial effect compared to that of viscous effect. Since ${\text{Re}} = \frac{{ca^{2} }}{{\nu_{f} }}$ so for ${\text{Re}}$ = 0, the stretching rate $c$ tends to vanishing since the cylinder radius $a$ cannot be zero in the present case. Also, the thickness is made infinite for finite deposition rate and the steady form cannot exist.

**Figure 8:**
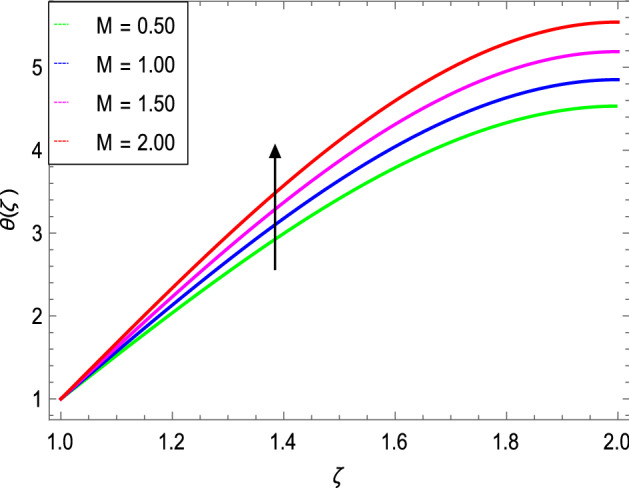
*θ*(ζ) as a function of *M*.

**Figure 9:**
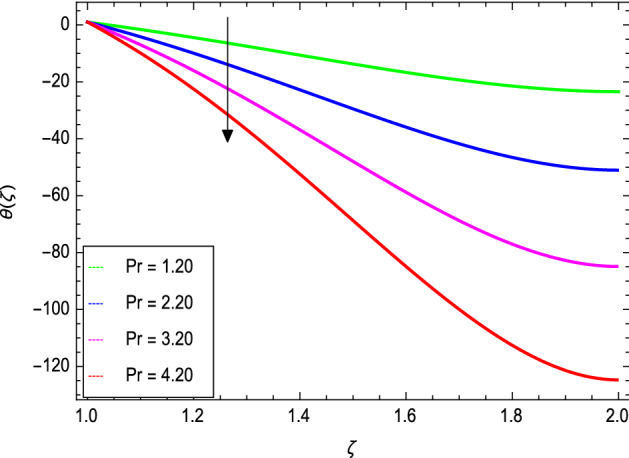
*θ*(ζ) as a function of *Pr*.

**Figure 10:**
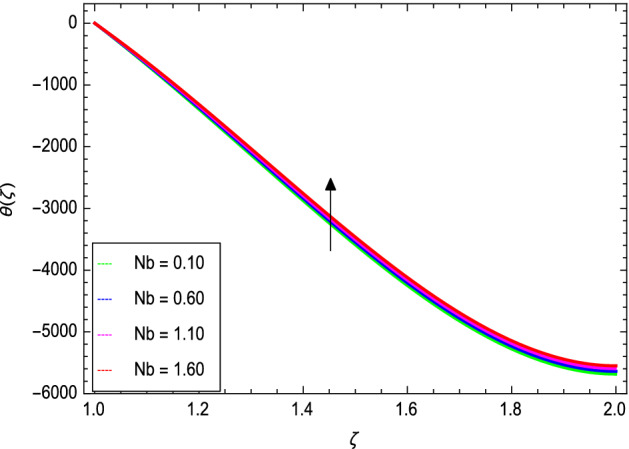
*θ*(ζ) as a function of *Nb*.

**Figure 11:**
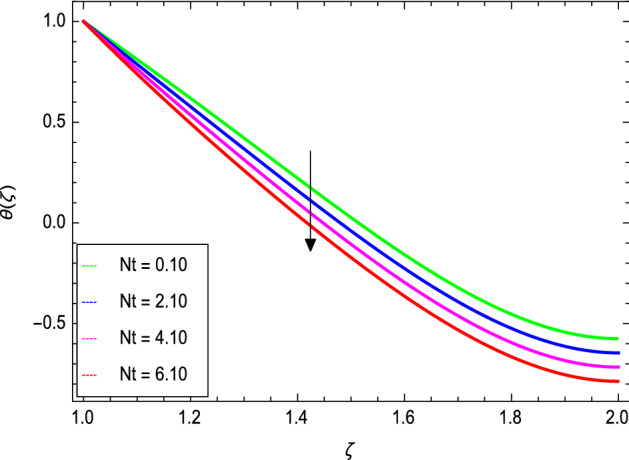
*θ*(ζ) as a function of *Nt*.

**Figure 12:**
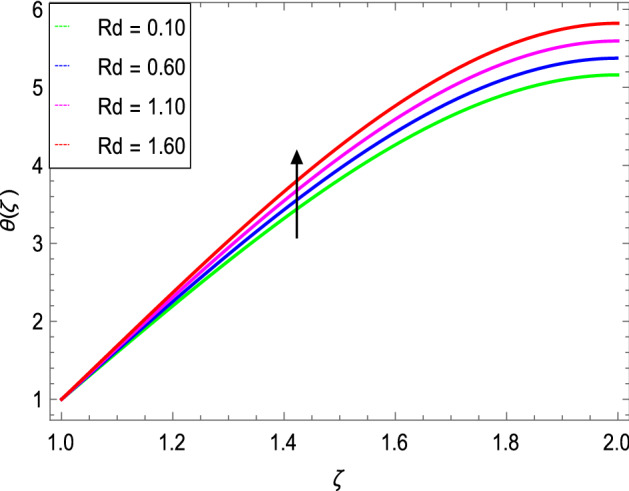
*θ*(ζ) as a function of *Rd*.

**Figure 13:**
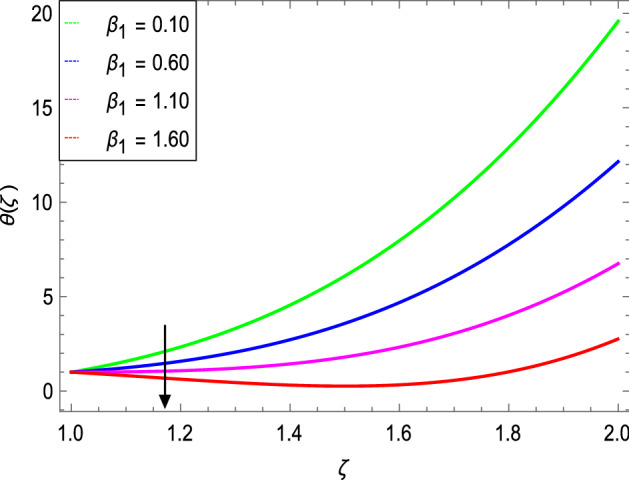
*θ*(ζ) as a function of *β*1.

Figures 8 and 9 depict the effects of the magnetic field and Prandtl number on the temperature profile. Figure 8 reveals that increasing the values of the magnetic parameter ${\text{M}}$, increases the temperature of the nanofluid. The magnetic field produces a resistive force that opposes the flow field and increases the thickness of the thermal boundary layer, consequently heat transfer increases. Figure 9 shows that the nanofluid temperature drops when the values of $\Pr$ increases, thus the thermal boundary layer decreases for higher quantities of $\Pr$ which shows that the effective cooling for nanofluid is achieved quickly. Given the relatively small size of the motion layer, the influence of a high Prandtl number is even clearer. The liquid retains a low thermal boundary layer for larger amounts of $\Pr$ which leads to a thinner thermal boundary layer resulting in an increase in heat transfer rate on the surface. Figures 10 and 11 show the effects of the Brownian motion parameter $Nb$ and the thermophoresis parameter $Nt$ on the temperature profile. Figure 10 shows that the enhancement in temperature of the fluid is observed with the increasing values of $Nb$ which results in decrease in the friction of the free surface of nanoparticles. Figure 11 shows that the temperature of nanofluid decreases as the $Nt$ values increase**.** Thermophoresis is a phenomenon of the diffusion of particles because of a temperature gradient effect. The force that transfers nanoparticles to the ambient fluid due to the temperature gradient is called thermophoretic force. Increased thermophoretic force results in a wider transfer of nanoparticles to the fluid layer. Figures 12 and 13 show the impacts of thermal radiation parameter $Rd$ and film thickness parameter $\beta_{1}$ respectively on the temperature profile. As shown in Fig. 12, the radiation parameter is used to add heat to the temperature of the nanoparticles as the temperature of the nanofluid rises. The analysis of thermal radiation is essential in the cooling of the cylinder. The thin film parameter $\beta_{1}$ has a special role in the temperature distribution. The temperature of the thermal boundary surface is high and small along with the transverse distance. The film thickness parameter, as shown in Fig. 13, reduces the temperature for greater values. The heat transfer rate is improved by thinning the nanofluid. In the present case, however, it is depreciating. The reason for this is that as the thickness of the fluid film increases, so does the mass of the fluid, which exhausts the temperature. As a result, heat enters the fluid and the environment cools. Thick film fluid requires more heat than thin film fluid.

**Figure 14:**
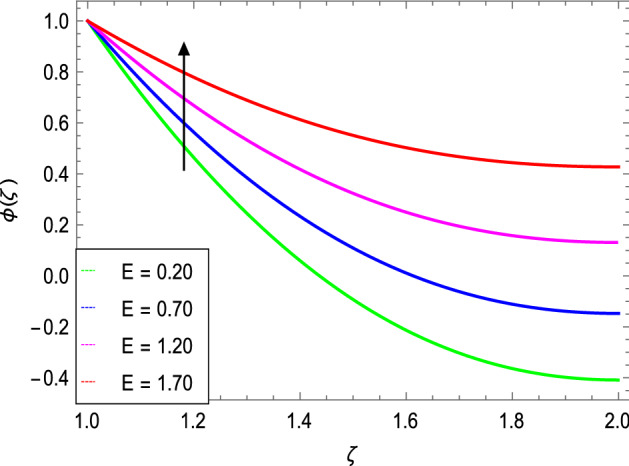
*ϕ*(ζ) as a function of *E*.

**Figure 15:**
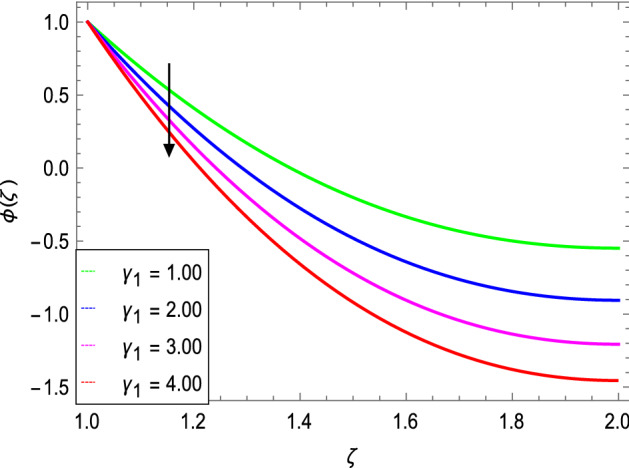
*ϕ*(ζ) as a function of *γ*_*1*_.

**Figure 16:**
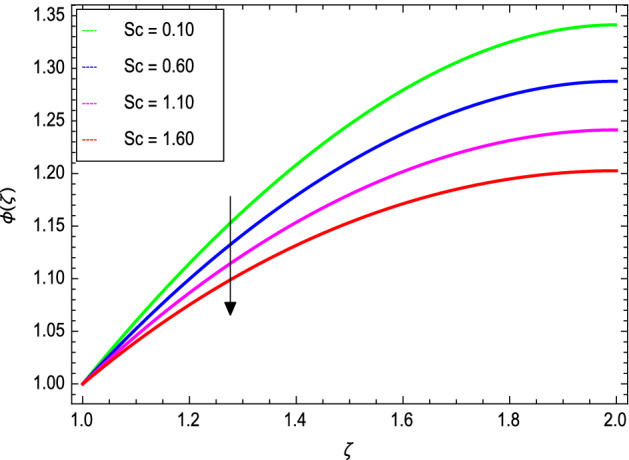
*ϕ*(ζ) as a function of *Sc*.

Figures 14 and 15 portray the influence of the activation energy parameter $E$ and the binary chemical reaction parameter $\gamma_{1}$ on the concentration profile and show that it is incremented with larger values of E while it is decreased with enlarging values of $\gamma_{1}$ respectively. The effect of Schmidt number $Sc$ on the nanoparticle’s concentration profile is presented in Fig. 16. The Schmidt number $Sc$ is related to the mass diffusions and therefore increases the mass diffusivity values leading to lessen down the nanoparticle’s concentration due to the less mass diffusion transportation as observed in Fig. 16.

**Figure 17:**
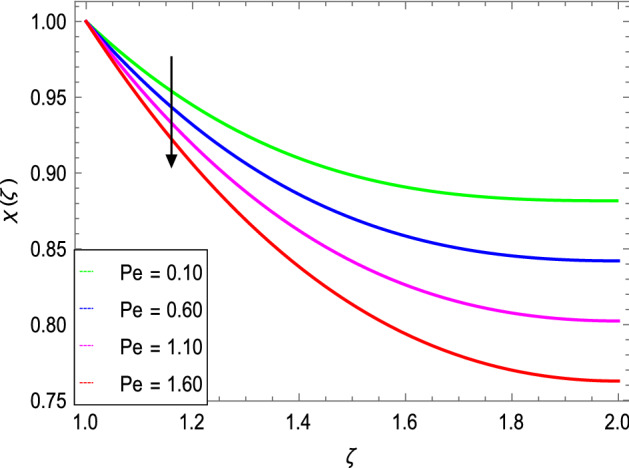
*χ*(ζ) as a function of *Pe*.

**Figure 18:**
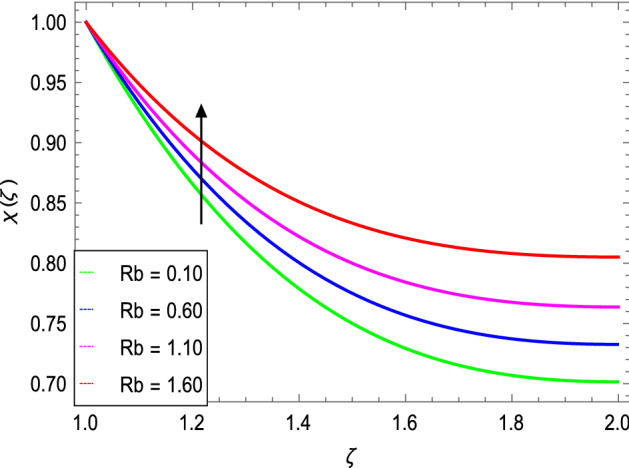
*χ*(ζ) as a function of *Rb*.

Figure 17 manifests the influence of Peclet number $Pe$. It shows a decrement in the boundary layer thickness of the motile microorganisms. The maximum values of $Pe$ result a fall in the diffusivity of the microorganisms. Figure 18 portrays the influence of $Rb$ on motile microorganism’s density. It shows that $\chi \left( \varsigma \right)$ increases with increasing the bioconvection Rayleigh number. The density of motile microorganisms is higher than that of liquid (water) and generally swims upwards to the outside (wall) of the cylinders.

**Figure 19:**
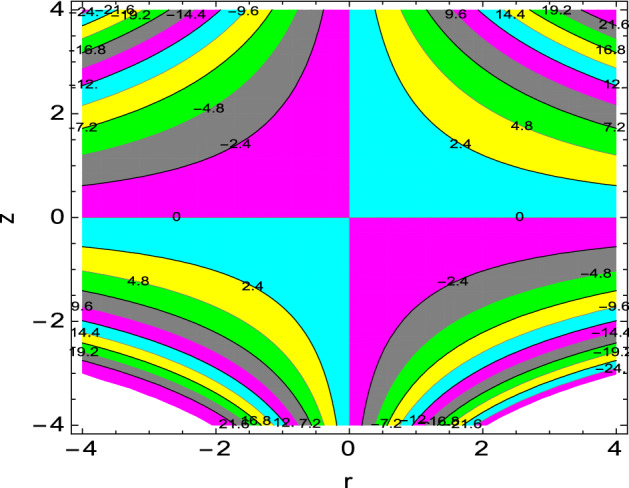
Streamlines for *M* = 0.30.

The streamlines are the tangent curves to the local instantaneous velocity field. The formation of an inner mixing bolus within a fluid surrounded by streamlines is referred to as trapping. Figure 19 depicts the effect of the magnetic field parameter on the streamlines. It is shown that the number of the trapped boluses increases when the value of magnetic field parameter $M$ is 0.30 which shows that the flow velocity is highly influenced by the magnetic field. The compression of streamlines is high at the lower portion compared to that of upper portion at the surface of stretching cylinder.

**Figure 20:**
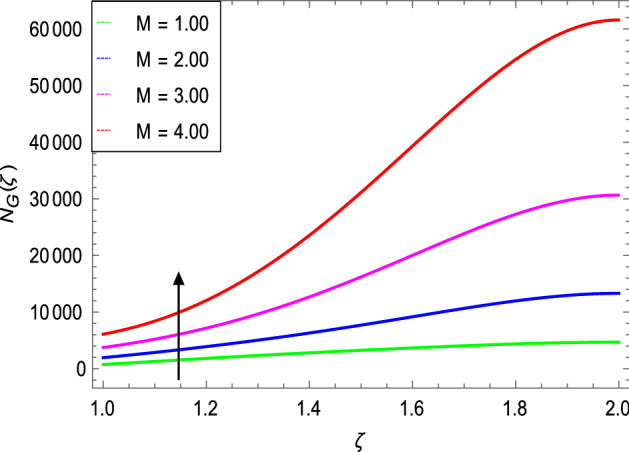
*NG*(ζ) as a function of *M*.

Figure 20 shows that the entropy generation increases as the magnetic field parameter increases. In general, increasing the magnetic field parameter causes a slight increase in entropy generation. Because the magnetic parameter has little influence on entropy generation, a wide difference in the magnetic field parameter results in a small variation in entropy.

## Comparison of the present work with published work

The present work is compared with the published work^79^ in Table 1 for various values of Oldroyd-B nanofluid parameter which shows the close agreement. In Tables 2, 3, 4 and 5, the different profiles show the different values including maximum and minimum for different parameters.

**Table 1:** Comparison of the present research with published paper for $- f^{\prime\prime}(0)$.

$\lambda_{1}$	Published work^80^	Present study
0.0	1.000000	1.000000
0.2	1.0518899	1.0518799
0.4	1.1019033	1.1019133

**Table 2:** Variation in skin friction coefficient $- f^{\prime\prime}(1)$ for $M,\lambda_{1} ,\lambda_{2} ,Gr{\text{ and }}Gm.$

$M$	$\lambda_{1}$	$\lambda_{2}$	$Gr$	$Gm$	$- f^{\prime\prime}\left( 1 \right)$
0.2	0.3	0.3	0.5	0.5	0.4231
0.6					0.3231
1.0					0.2231
0.5	0.1				0.1231
	0.4				0.1231
	0.7				0.0231
	0.3	0.2			0.1031
		0.4			0.1031
		0.6			0.1231
		0.3	0.1		0.0231
			0.6		0.0231
			1.0		0.1230
			0.5	0.1	0.1201
				1.0	0.1031
				2.0	0.0031
				0.5	0.1031

**Table 3:** Variation in Nusselt number $- \theta ^{\prime}(1)$ for $M,\lambda_{1} ,\lambda_{2} ,Gr,Gm,\Pr ,Nb,Nt,Lb{\text{ and }}Rd.$

$M$	$\lambda_{1}$	$\lambda_{2}$	$Gr$	$Gm$	$\Pr$	$Nb$	$Nt$	$Lb$	$Rd$	$- \theta ^{\prime}\left( 1 \right)$
0.5	0.3	0.3	0.5	0.5	2.0	0.3	0.3	5.0	0.8	0.2764
0.2										0.2763
0.6										0.2755
1.0										0.2745
	0.1									0.2735
	0.4									0.2725
	0.7									0.2715
		0.2								0.2754
		0.4								0.2753
		0.6								0.2760
			0.1							0.2761
			0.4							0.2762
			0.7							0.2736
				0.1						0.2735
				1.0						0.2734
				2.0						0.2733
					0.1					0.2732
					1.0					0.2731
					2.0					0.2730
						1.0				0.2729
						3.0				0.2728
						5.0				0.2727
							0.1			0.2726
							0.4			0.2725
							0.8			0.2724
								1.0		0.2723
								2.0		0.2722
								3.0		0.2721
									0.1	0.2720
									0.3	0.2717
									0.5	0.2716

**Table 4:** Variation in Sherwood number $- \phi ^{\prime}(1)$ for $E,\lambda_{1} ,\lambda_{2} ,Gr,Gm,\Pr ,Nb,Nt,Le{\text{ and }}E.$.

$M$	$\lambda_{1}$	$\lambda_{2}$	$Gr$	$Gm$	$\Pr$	$Nb$	$Nt$	$Le$	$E$	$- \phi ^{\prime}\left( 1 \right)$
0.5	0.3	0.3	0.5	0.5	2.0	0.3	0.3	5.0	0.1	0.15479
0.2										0.15478
0.6										0.15477
1.0										0.15476
	0.1									0.15475
	0.4									0.15474
	0.7									0.15473
		0.2								0.15472
		0.4								0.15471
		0.6								0.15470
			0.1							0.15469
			0.6							0.15468
			1.0							0.15467
				0.1						0.15468
				1.0						0.15467
				2.0						0.15466
					1.0					0.15465
					3.0					0.15464
					5.0					0.15463
						0.1				0.15462
						0.3				0.15461
						0.5				0.15460
							0.1			0.15459
							0.4			0.15458
							0.8			0.15457
								1.0		0.15456
								2.0		0.15455
								3.0		0.15454
									0.5	0.15453
									1.0	0.15452
									1.5	0.15451

**Table 5:** Variation in motile microorganism density number $- \chi ^{\prime}(1)$ for $M,\lambda_{1} ,\lambda_{2} ,Gr,Gm,Lb{\text{ and }}Pe.$

$M$	$\lambda_{1}$	$\lambda_{2}$	$Gr$	$Gm$	$Lb$	$Pe$	$- \chi ^{\prime}\left( 1 \right)$
0.5	0.3	0.3	0.5	0.5	1.0	0.1	0.3989
0.6							0.3988
1.0							0.3987
	0.1						0.3986
	0.4						0.3985
	0.7						0.3984
		0.2					0.3983
		0.4					0.3982
		0.6					0.3981
			0.1				0.3980
			0.6				0.3979
			1.0				0.3978
				0.1			0.3977
				1.0			0.3976
				2.0			0.3975
					0.1		0.3974
					0.5		0.3973
					0.8		0.3972
						0.2	0.3971
						0.6	0.3970
						1.0	0.3969

## Conclusions

The heat and mass transfer flow of an Oldroyd-B nanoliquid film sprayed on a stretching cylinder containing gyrotactic microorganisms is investigated using similarity transformations. Thermodynamics and spraying phenomena are mathematically modeled and then analyzed using HAM solution with profiles such as spray rate, velocity, heat and mass transfer, and gyrotactic microorganism’s motion.

The summary of findings are as follows:

- Spray rate increases with the film thickness nonlinearly.
- The velocity profile shows decreasing behavior for magnetic field parameter, bioconvection Rayleigh number and Oldroyd-B nanofluid parameter while increases with thermal Grashof, solutal Grashof and Reynolds numbers.
- The temperature increases with increasing the magnetic field, Brownian motion and thermal radiation parameters while it is decreased with the positive values of Prandtl number, film thickness and thermophoresis parameters.
- The concentration profile shows an increasing behavior with the activation energy parameter while it decreases with increasing the thermal radiation, chemical reaction parameter and Schmidt number as well.
- The gyrotactic microorganisms motion increases with increasing the bioconvection Rayleigh number while it is decreased with the Peclet and Lewis numbers.
- The entropy generation increases with the magnetic field parameter.
- Skin friction coefficient, heat and mass transfer rate, and motile density number consistently decrease with the different parameters.

## Data Availability

Availability exists for the data upon request.

## Abbreviations

$$b_{1}$$: Chemotaxis constant
$$b$$: Outer radius
$$C$$: Nanoparticles concentration
$C_{w}$: Nanoparticles concentration at the wall
$C_{b}$: Nanoparticles concentration beyond the surface
$B_{o}$: Magnetic field strength (N A^−^^1^ m^−^^1^)
$C_{f}$: Skin friction coefficient
$D_{B}$: Brownian diffusion coefficient (m^2^ s^−^^1^)
$$D_{m}$$: Mass diffusivity (m^2^ s^−^^1^)
$D_{T}$: Thermophoretic diffusion coefficient (m^2^ s^−^^1^)
$E$: Arrhenius activation energy parameter
$E^{\prime\prime\prime}_{0}$: Dimensional characteristic entropy generation
$E^{\prime\prime\prime}_{gen}$: Dimensional entropy generation
$f$: Dimensionless velocity function
$g$: Acceleration due to gravity (m s^−^^2^)
$Gr$: Thermal Grashof number
$Gm$: Solutal Grashof number
$k$: Thermal conductivity parameter
$k_{0}$: Relaxation time coefficient
$k_{1}$: Retardation time coefficient
$$k^{**}$$: Mean absorption coefficient
${\text{Lb}}$: Bioconvection Lewis number
$M$: Magnetic field parameter
$N$: Motile density of microorganisms
$N_{b}$: Motile density of microorganisms beyond the surface
$N_{w}$: Motile density microorganisms at the wall
$Nb$: Dimensionless Brownian motion parameter
$N_{G} (\varsigma )$: Dimensionless entropy generation
$Nt$: Dimensionless thermophoresis parameter
$Nu$: Local Nusselt number
$$P$$: Pressure (kg m^−^^1^ s^−^^2^)
$${\text{Pe}}$$: Peclet number
$$\Pr$$: Prandtl number
$q_{rz}$: Wall heat flux
$q_{h}$: Wall mass flux
$q_{n}$: Wall density flux
$Rb$: Bioconvection Rayleigh number
${\text{Re}}_{x}$: Local Reynolds number
$Rd$: Thermal radiation parameter
$$Sc$$: Schmidt number
$Scb$: Bioconvection Schmidt number
$$Sh$$: Sherwood number
$Sn$: Local motile microorganism density number
$T$: Temperature of the fluid (K)
$T_{b}$: Temperature at the outer radius of the film surface (K)
$T_{w}$: Temperature at the wall (K)
$$u, \, w$$: Velocity components (m s^−^^1^)
$V$: Radial axisymmetric spray velocity (m s^−^^1^)
$W_{c}$: Speed of gyrotactic cell

## Greek symbols

$\zeta$: Dimensionless similarity variable
$\rho_{f}$: Density of nanofluid (kg m^−^^3^)
$\rho_{m}$: Motile microorganism density (kg m^−^^3^)
$\rho_{p}$: Density of nanoparticles (kg m^−^^3^)
$\lambda_{1}$: Deborah number on behalf of relaxation time
$\lambda_{2}$: Deborah number on behalf of retardation time
$\gamma_{1}$: Chemical reaction rate
$$\sigma$$: Electrical conductivity (S m^−^^1^)
$$\sigma^{**}$$: Stefan–Boltzmann constant (J K^−^^1^)
$$\mu_{f}$$: Dynamic viscosity (N s m^−^^2^)
$$v_{f}$$: Kinematic viscosity (m^2^ s^−^^1^)
$$\alpha_{1}$$: Thermal diffusivity(m^2^ s^−^^1^)
$\beta_{1}$: Nondimensional film thickness parameter
$\chi$: Dimensionless motile microorganism’s concentration
$\phi$: Dimensionless nanoparticles concentration
$$\theta$$: Dimensionless temperature
$\left( {\rho c_{p} } \right)_{f}$: Heat capacity of nanofluid (J K^−^^1^)
$\left( {\rho c_{p} } \right)_{p}$: Heat capacity of nanoparticle (J K^−^^1^)
$\tau$: Ratio of heat capacity
$\tau_{w}$: Wall shear stress
